# Validation of the 7-Item Domain-General Gambling Harm Scale (DGHS-7)

**DOI:** 10.1016/j.abrep.2023.100499

**Published:** 2023-06-02

**Authors:** André Syvertsen, Joakim H. Kristensen, Matthew Browne, En Li, Ståle Pallesen

**Affiliations:** a Department of Psychosocial Science, University of Bergen, Norway; b Norwegian Competence Center for Gambling and Gaming Research, University of Bergen, Norway; c Central Queensland University, Australia

**Keywords:** Gambling consequences, Gambling measure, Harm screen, Problem gambling, Scale validation

## Abstract

**Introduction:**

Gambling can cause negative consequences affecting finances, work/study, physical and mental health, relationships, law abidingness, and the community. Although existing measures enable investigations of gambling harms, there is still a need for a brief measure covering the full range of gambling related harms.

**Methods:**

We validated a 7-item domain-general harm scale (DGHS-7) using data from a cross-sectional survey of United Kingdom residents reporting gambling within the last 12 months (*n* = 2558, 62.4% women, mean age 40.1 years (*SD* = 12.5)). The DGHS-7 was investigated in terms of factor structure, measurement invariance, and convergent validity with a comprehensive 72-item checklist of gambling harm, the Short Gambling Harms Screen (SGHS), and the Problem Gambling Severity Index (PGSI). Discriminative validity was checked against the Personal Wellbeing Index (PWI). Internal consistency was also calculated.

**Results:**

Confirmatory factor analysis supported a one-factor solution (χ^2^ = 136.991, *df* = 14, χ^2^/*df* = 9.785, *p* <.001, CFI = 0.999, RMSEA = 0.059, 90% CI [0.050, 0.068]). Measurement invariance was supported for gender and binary categorization of age and income (ΔCFI = 0.001). The DGHS-7 correlated strongly with the 72-item checklist (*r_s_* = 0.824), the SGHS (*r_s_* = 0.793), the PGSI (*r_s_* = 0.768), and moderately with the PWI (*r_s_* = -0.303). Cronbach’s alpha = 0.91 and ordinal alpha = 0.96 indicated good internal consistency.

**Conclusions:**

Psychometric support was found for a brief measure covering all recognized domains of gambling harm. The DGHS-7 is useful for researchers needing a generic and short measure for epidemiological and other studies calling for short scales.

## Highlights

• Gambling can negatively affect multiple life domains.
• Accurate measurement is important for preventing gambling harm.
• The DGHS-7 shows robust psychometric properties while measuring all harm domains.
• The DGHS-7 is especially useful for studies facing challenges with survey length.

## 1 Introduction

Gambling can constitute an unproblematic and enjoyable activity for individual consumers. In contrast, harmful gambling is associated with negative health and wellbeing impacts for the gambler, others connected to the gambler, and/or the wider community, making this a public health concern (Shaffer & Korn, 2002). Negative consequences from gambling are especially frequent among individuals with some degree of gambling problems, a concept that also encompasses symptoms of behavioral dependence, such as pre-occupation with gambling, chasing losses, lack of control while gambling, and increasing involvement (Potenza et al., 2019). Problem gambling is estimated to affect between 0.12% and 5.8% worldwide (Calado & Griffiths, 2016). However, problem gambling estimates do not fully reflect the range of harms that arise from gambling because negative consequences from gambling can also occur in the absence of the distinct symptoms associated with problem gambling (Browne et al., 2021). Gambling harm can conceivably occur across the full spectrum of gambling involvement and the majority of harm at the aggregated level appears concentrated among those classified as having a low or moderate risk for problem gambling, because of the much larger number of gamblers in this subclinical category (Browne & Rockloff, 2018). Further, most specific problem gambling measures only capture a selection of harms because they are anchored within disordered gambling classification rather than assessing a wider range of negative consequences from gambling per se (Shannon et al., 2017). Consequently, on both conceptual and pragmatic grounds, a complete understanding of the negative consequences of gambling in a society requires that gambling harm is measured independently from problem gambling.

Gambling harm has been categorized and assessed in various ways (Browne et al., 2021). Notably, items from problem gambling measures have frequently been repurposed to measure gambling harm, although there are now increasing efforts to develop more dedicated indicators of gambling harm. Langham et al. (2016) developed a taxonomy for gambling harm informed by a literature review, focus groups and interviews with health professionals, interviews with people experiencing gambling problems and affected-others, as well as forum posts on problem gambling. This work led to a categorization of seven domains of gambling harm: Financial harms, work/study harms, health harms, emotional/psychological harms, relationship harms, cultural harms, and crime activity (Langham et al., 2016).

Langham et al., (2016)’s taxonomy of gambling harm has been measured by the comprehensive 72-item checklist for gambling harm (Li et al., 2017). Due to the extensive nature of the 72-item checklist, the Short Gambling Harm Screen (SGHS) was later developed to serve as a more practical measure of general population levels of gambling harm (Browne et al., 2018). The SGHS was designed to maximize sensitivity to capture a broad spectrum of gambling harm. It shows robust psychometric properties, including external validity when comparing it to measures of problem gambling such as the Problem Gambling Severity Index (PGSI) (Ferris & Wynne, 2001). The SGHS has since been validated and applied in several studies (Browne et al., 2021, Murray Boyle et al., 2021, Murray Boyle et al., 2022). However, the intention to maximize sensitivity resulted in the 10-item SGHS having items tapping only 3 out of the 7 domains of gambling harm. Furthermore, 5 of the 10 items reflect the financial domain, 4 reflect the emotional/psychological domain, and 1 reflects the relationship domain. Consequently, while the SGHS constitutes a psychometrically good measure of the unidimensional latent factor of general gambling harm, it does not provide representative coverage of all dimensions identified by Langham et al. (2016). Recently, an 18-item version of the SGHS was made to provide a shorter version of the 72-item checklist that could still cover all gambling domains (Latvala et al., 2021). This 18-item version of the SGHS shows good convergent and external validity, as well as reliability.

Brief gambling harm measures can be especially useful for population-level studies with epidemiologic purposes that use surveys with limited length. Response rates to surveys are falling and reducing survey length is increasingly important for combatting non-response (Edwards et al., 2009, Stedman et al., 2019). Following this, it appears beneficial to examine if a 7-item gambling scale that covers all domains can serve as a satisfactory measure of gambling harm. Such a measure could also be more practical to include in studies that focus on multiple topics simultaneously such as broad epidemiological surveys. Finally, the approach of prior harms scales has been to tap concrete, but very specific, symptomatology from the full 72 item checklist. Because specific harms tend to be somewhat idiosyncratic, this might cause the scales to suffer if individuals are experiencing differing symptoms. Accordingly, the present study aimed to assess the psychometric properties of a broader ‘domain-focused’ 7-item scale, including its factor structure, convergent validity, discriminative validity, and internal consistency. However, as the domain-focused scale covers harm categories rather than specific harms with descriptions, it was important to assess whether the presence or absence of such descriptions and their order of presentation influence overall levels of reported harms. This was investigated with a survey experiment detailed below. The present study also assessed the 7-item scale for measurement invariance for age, gender, and income groups.

## 2 Material and methods

### 2.1 Participants and procedure

The current study was based on data from a cross-sectional survey administered to individuals from the United Kingdom in April 2022. The survey was developed using the online survey tool SurveyXact (surveyxact.com) and administered through the recruitment service for online participants Prolific (prolific.co). Recruitment was continued until 3000 valid responses were received, which resulted in a sample of 64.0% women and a mean age of 40.0 years (*SD* = 12.6). Participants were included for further data analysis if they reported any gambling activity within the last 12 months, resulting in a final sample of *n* = 2558, consisting of 62.4% women and a mean age 40.1 years (*SD* = 12.5). The mean time to complete the full survey (among those who had gambled the last 12 months) was 8.5 min (*SD* = 7.2), and participants were compensated by 2.5£. We also included a yes/no response attention-check item (“The current head of state in the UK is a king”) which was passed by all respondents. The study was exempt from ethical approval in accordance with the Norwegian Centre for Research Data's guidelines for anonymous surveys. Participants were regarded as having provided informed consent by completing the survey.

### 2.2 Measures

#### 2.2.1 Demographic information

Demographic measures included gender (male, female), age (year of birth), education, and income. Education was assessed with drop-down menu with 8 options: “No formal qualifications” (1), “Secondary education (e.g., GED/DCSE)” (2), “High school diploma/A-levels” (3), “Technical/community college” (4), “Undergraduate degree (BA/BSc/other)” (5), “Graduate degree (MA/MSc/MPhil/other)” (6), “Doctorate degree (PhD/other)” (7), and “Don’t know / not applicable” (8). Income after tax was assessed with drop-down menu with 11 options: (1) constituted “Less than £10,000”, (2) to (10) constituted intervals increasing with £10,000, and (11) constituted “More than £100,000”.

#### 2.2.2 Problem gambling

Problem gambling was assessed with the PGSI (Ferris & Wynne, 2001). The PGSI is a validated scale for problem gambling containing four items assessing problematic gambling behavior (e.g., “have you borrowed money or sold anything to gamble?”) and five items assessing negative consequences from gambling (e.g., “has your gambling caused any financial problems for you or your household?”). The items are scored on a 4-point scale from 0 (“never”) to 3 (“always”). Responses are categorized by a composite score in which non-problem = 0, low risk = 1–2, moderate risk = 3–7, and problem gambling = 8–27, a range of 0–27. Cronbach’s alpha was 0.91 (*n* = 2558) in the current study.

#### 2.2.3 Personal wellbeing

Wellbeing was assessed by the personal wellbeing index (PWI) (Cummins et al., 2003, Lau et al., 2005). The PWI is a validated scale for well-being containing seven items assessing wellbeing across life domains, including: Standard of living, health, achievements, personal relationships, safety, sense of community, and sense of future security. Items are scored on an 11-point scale from 0 (“no satisfaction at all”) to 10 (“completely satisfied”). One example item is “how satisfied are you with your standard of living?”. Scores are summed across each item and divided by the number of items (7) and multiplied by 10 to produce a well-being score between 0 and 100. Cronbach’s alpha was 0.91 (*n* = 2558) in the current study.

#### 2.2.4 Gambling harm

Gambling harm was measured by the 72-item checklist for gambling harms, which also contains the 10 items constituting the SGHS (Browne et al., 2018, Langham et al., 2016, Li et al., 2017). The 72-item checklist is a comprehensive measure of gambling harm based on the taxonomy reported in Langham et al. (2016). Li et al. (2017) translated these gambling harms into plain language personal statements with binary yes/no response option. Respondents are asked to reflect on whether gambling has caused specific harms, for example “felt ashamed of my gambling” (emotional/psychological) and “increased credit card debt” (financial). Additionally, the 72-item checklist contains a 4-point item for each gambling harm domain covering overall gambling harm experienced within the financial, work/study, health, emotional/psychological, relationship, and other domains. The other category represents a combination of cultural harms and harms relevant to criminal activity (i.e., law-abidingness). Responses range from “no impact” (0) to “major impact” (3). In the current study, these domain-general items were modified to function better independently: Each response alternative was expanded to a 5-point scale, and the “other” category was separated back into the two categories cultural and law-abidingness harms consistent with Langham et al. (2016)’s original taxonomy. See appendix and Table 3 for the full version of this domain-general gambling harm (DGHS-7) scale. The ranges of possible sum scores are 0–72 for the 72-item checklist, 0–10 for the SGHS, and 0–28 for the DGHS-7.

### 2.3 Statistical analysis

Descriptive statistics included distribution of participant age, gender, education level, income bracket, PGSI category, and proportion having experienced any gambling harm.

Previous investigation of the 72-item checklist and the SGHS shows a unidimensional structure, suggestive of one latent factor for gambling harm (Browne et al., 2018). Thus, we conducted a confirmatory factor analysis (CFA) based on the 7 domain general items to check whether there was support of a unidimensional structure. We used weighted least squares means and variances (WLSMV) as these are designed for ordinal level data, leading to better estimation of factor loadings and handling of non-normality of observed variables (Li, 2016). Model fit was assessed with root mean square error of approximation (RMSEA) where value ≤ 0.06 indicates good fit and comparative fit index (CFI) where value ≥ 0.95 constitutes good fit (Hu & Bentler, 1999). Measurement invariance was examined for age, gender, and income bracket, following Wu & Estabrook’s (2016) approach for ordinal data. Age and gender appear as robust correlates of problem gambling and age and gender comparisons for gambling harm thus necessitate information on (lack of) measurement invariance (Allami et al., 2021). Measurement invariance across income brackets informs comparisons in gambling harm between individuals with varying purchasing power. Age was categorized into groups 18–29, 30–49, and 50+, similar to Browne et al. (2018). Income was categorized with median split (Below 20,000–29,000£ versus 20,000–29,000£ and above) due to low variance at high income brackets. Configural invariance across groups was investigated by estimating the same model for each group separately and assessing model fit (RMSEA, CFI). Threshold, metric, scalar, and residual invariance was then investigated in a stepwise fashion by comparing pairs of models with restrictions to thresholds, loadings, intercepts, and residuals. Each model was compared to their less restricted counterpart. We followed reporting recommendations by Putnick & Bornstein (2016). Delta CFI values ≤ 0.01 in model comparisons were taken to support measurement invariance as chi-square difference tests are susceptible to sample size (Cheung and Rensvold, 2002, Putnick and Bornstein, 2016).

Reliability was assessed with Cronbach’s alpha, ordinal alpha, and McDonald’s omega. Ordinal alpha may estimate reliability more accurately than Cronbach’s alpha on ordinal items and coefficient omega (hierarchical) allows for assessing unidimensionality (Gadermann et al., 2012, Zinbarg et al., 2005). Values range from −1 to 1 and the established cutoff from Cronbach’s alpha was utilized for the three coefficients, in which ≥ 0.70 indicates acceptable reliability. We also estimated reliability coefficients (Cronbach) if a specific item was dropped.

Convergent validity for the proposed 7-item domain general gambling harm scale (DGHS-7) was assessed against the 72-item checklist for gambling harm, the SGHS, and the PGSI, and discriminant validity against the PWI. We calculated Spearman correlations between composite scores on each measure. Finally, mean scores on the PGSI and PWI were compared between participants who scored zero and those scoring higher than zero on the DGHS-7 with two-tailed independent samples *t*-tests and effect size Cohen’s *d.* This was done to examine whether experiencing any gambling harm would be associated with more problem gambling and less well-being, compared to not experiencing any gambling harm at all. Finally, we visualized average PGSI and PWI scores by groups of DGHS-7 scores to examine if minor increases in gambling harm were associated with less well-being and more problem gambling. Local polynomial regression fitting was applied to individual sum scores.

The present study also examined if the presentation order of the DGHS-7 and 72-item checklist for gambling harm (i.e., DGHS-7 first vs. 72-item checklist first) could result in different mean values on specific DGHS-7 items and/or its composite score.

Descriptive statistics and CFA were conducted using the statistical program R version 4.2.1 with *gtsummary* package version 1.6.1 and *lavaan* package version 0.6–11, respectively (Rosseel, 2012, Sjoberg et al., 2021). The remaining analyses were conducted with IBM SPSS Version 28.

## 3 Results

Participant characteristics are reported in Table 1. CFA was conducted on data from participants who reported gambling the last 12 months (*n* = 2558) and indicated good fit in terms of a one-factor solution of gambling harms (χ^2^ = 136.991, *df* = 14, χ^2^/*df* = 9.785, *p* <.001, CFI = 0.999, RMSEA = 0.059, 90% CI [0.050, 0.068]). Models could not be compared for age group categorization of 18–29, 30–49, and 50 + due to low variance in “Law-abidingness” item in the 18–29 group (no one in the 18–29 group reported “major impact (4)”). Due to this we opted for an alternative approach with median split of age (below 38 years versus 38 years or above). Full results on measurement invariance tests are reported in Table 2 and indicate configural, threshold, metric, scalar, and residual invariance for gender, median split for age, and median split for income according to delta CFI value ≤ 0.01 threshold. Distribution of responses on the DGHS-7 is presented in Table 3. Results on reliability analyses for the DGHS-7 showed that Cronbach’s alpha reliability was 0.91, Ordinal alpha was 0.96, and McDonald’s omega (hierarchical) was 0.82 indicating good reliability. Reliability if an item was dropped is presented in Table 3 and ranged between 0.88 and 0.92.

**Table 1:** Participant Characteristics (n = 2,558).

Characteristic	
Age, *M* (*SD*)	40.1 (12.5)
Women, *n* (%)	1,597 (62%)
*Education level, n (%)*	
No formal qualifications	14 (0.5%)
Secondary education	306 (12%)
High school diploma/A-levels	449 (18%)
Technical/Community college	320 (13%)
Undergraduate degree	1,018 (40%)
Graduate degree	405 (16%)
Doctorate degree	44 (1.7%)
Don’t know / not applicable	2 (<0.1%)
*Net income bracket, n (%)*	
Less than £10,000	406 (16%)
£10,000 - £19,999	547 (21%)
£20,000 - £29,999	725 (28%)
£30,000 - £39,999	440 (17%)
£40,000 - £49,999	204 (8.0%)
£50,000 - £59,999	104 (4.1%)
£60,000 - £69,999	56 (2.2%)
£70,000 - £79,999	35 (1.4%)
£80,000 - £89,999	17 (0.7%)
£90,000 - £99,999	9 (0.4%)
More than £100,000	15 (0.6%)
*PGSI category, n (%)*	
Non-problem	1,139 (45%)
Low risk	695 (27%)
Moderate risk	506 (20%)
Problem gambling	218 (8.5%)
Any gambling harm, *n* (%)	1,213 (47%)

Note. PGSI = Problem Gambling Severity Index.

**Table 2:** Results from confirmatory factor analyses.

					Base Model and Multi-Group Measurement Invariance Tests									
Models	*χ2 (*df)	CFI	TLI	RMSEA (90% CI)	RMR	SRMR	Model comparison	Δ*χ2* (Δdf)	ΔCFI	ΔTLI	ΔRMSEA	ΔRMR	ΔSRMR	Decision
Base model	136.991 (14)	0.999	0.999	0.059 (0.050, 0.068)	0.034	0.049	–	–	–		–		–	Accept
				Gender^a^										
M1a: Group configural model	153.987 (28)	0.999	0.999	0.059 (0.050, 0.069)	0.037	0.053	–	–	–		–		–	Accept
M2a: Threshold invariance	160.06 (42)	0.999	0.999	0.047 (0.039, 0.055)	0.047	0.053	M1a	13.954 (14)	<0.001	<0.001	-0.012	0.010	<0.001	Accept
M3a: Metric invariance	175.88 (48)	0.999	0.999	0.046 (0.039, 0.053)	0.058	0.053	M2a	18.298 (6)*	<0.001	<0.001	-0.001	0.011	<0.001	Accept
M4a: Scalar invariance	186.66 (54)	0.999	0.999	0.044 (0.037, 0.051)	0.060	0.053	M3a	8.775 (6)	<0.001	<0.001	-0.002	0.002	<0.001	Accept
M5a: Residual invariance	192.88 (61)	0.999	0.999	0.041 (0.035, 0.048)	0.058	0.053	M4a	4.788 (7)	<0.001	<0.001	-0.003	-0.002	<0.001	Accept
				Age (median split)^b^										
M1b: Group configural model	152.67 (28)	0.999	0.999	0.059 (0.050, 0.068)	0.035	0.050	–	–	–		–		–	Accept
M2b: Threshold invariance	161.08 (42)	0.999	0.999	0.047 (0.040, 0.055)	0.050	0.050	M1b	18.274 (14)	<0.001	<0.001	-0.012	0.015	<0.001	Accept
M3b: Metric invariance	161.46 (48)	0.999	0.999	0.043 (0.036, 0.050)	0.050	0.050	M2b	1.051 (6)	<0.001	<0.001	-0.004	<0.001	<0.001	Accept
M4b: Scalar invariance	167.35 (54)	0.999	0.999	0.041 (0.034, 0.048)	0.051	0.050	M3b	5.453 (6)	<0.001	<0.001	-0.002	0.001	<0.001	Accept
M5b: Residual invariance	218.52 (61)	0.999	0.999	0.045 (0.039, 0.051)	0.055	0.053	M4b	26.295 (7)**	<0.001	<0.001	0.004	0.004	0.003	Accept
				Income bracket (median split)^c^										
M1c: Group configural model	140.74 (28)	0.999	0.999	0.056 (0.047, 0.066)	0.035	0.050	–	–	–		–		–	Accept
M2c: Threshold invariance	148.14 (42)	0.999	0.999	0.044 (0.037, 0.052)	0.043	0.050	M1c	16.622 (14)	<0.001	<0.001	-0.012	0.008	<0.001	Accept
M3c: Metric invariance	154.71 (48)	0.999	0.999	0.042 (0.034, 0.049)	0.055	0.050	M2c	7.622 (6)	<0.001	<0.001	-0.003	0.012	<0.001	Accept
M4c: Scalar invariance	176.47 (54)	0.999	0.999	0.042 (0.035, 0.049)	0.058	0.050	M3c	16.447 (6)*	<0.001	<0.001	<0.001	0.003	<0.001	Accept
M5c: Residual invariance	211.19 (61)	0.999	0.999	0.044 (0.038, 0.050)	0.058	0.052	M4c	17.929 (7)*	<0.001	<0.001	0.002	<0.001	0.002	Accept

*Note.*^a^Women *n* = 1597, men *n* = 961. ^b^Below 38 years *n* = 1223, 38 years and above *n* = 1335. ^c^Below 20,000–29,000 £ *n* = 953, 20,000–29,000 £ and above *n* = 1605. Δχ2 (Δdf) statistics are adjusted according to Satorra (2000). *p ≤ 0.05. **p ≤ 0.001. CFI = Comparative Fit Index. TLI = Tucker-Lewis Index. RMSEA = Root Mean Square Error of Approximation. RMR = Root Mean Square Residual. SRMR = Standardized Root Mean Square Residual.

**Table 3:** Domain-General Gambling Harms Characteristics (*n* = 2558).

	Respone							
Item	“No impact” (0)	“Minor impact” (1)	“Some impact” (2)	“Moderate impact” (3)	“Major impact” (4)	Mean (SD)	Correlation item and scale^a^	Reliability if item is dropped^b^
What level of negative impact did your gambling have upon your financial security during this time?	1631 (64%)	667 (26%)	156 (6.1%)	72 (2.8%)	32 (1.3%)	0.52 (0.83)	0.79	0.89
What level of negative impact did your gambling have upon your personal relationships (family, friends, spouse, partner, etc.) during this time?	2094 (82%)	291 (11%)	100 (3.9%)	56 (2.2%)	17 (0.7%)	0.28 (0.70)	0.81	0.89
What level of negative impact did your gambling have upon your emotional or psychological wellbeing during this time?	1706 (67%)	521 (20%)	195 (7.6%)	91 (3.6%)	45 (1.8%)	0.53 (0.91)	0.85	0.89
What level of negative impact did your gambling have upon your physical or mental health during this time?	1881 (74%)	397 (16%)	153 (6.0%)	85 (3.3%)	42 (1.6%)	0.44 (0.87)	0.88	0.88
What level of negative impact did your gambling have upon your work or study performance during this time?	2131 (83%)	268 (10%)	104 (4.1%)	39 (1.5%)	16 (0.6%)	0.26 (0.66)	0.78	0.89
What level of negative impact did your gambling have upon your cultural or religious community during this time? (For example, feeling less connected or contributing less to cultural/religious community.)	2396 (94%)	102 (4.0%)	34 (1.3%)	20 (0.8%)	6 (0.2%)	0.10 (0.44)	0.53	0.91
What level of negative impact did your gambling have upon your law-abidingness during this time? (For example, taking money or items from friends or family without asking first.)	2443 (96%)	75 (2.9%)	27 (1.1%)	10 (0.4%)	3 (0.1%)	0.07 (0.35)	0.50	0.92

a Correlation of the item with the scale composed of the remaining items.

b Based on standardized alpha.

**Fig. 1:**
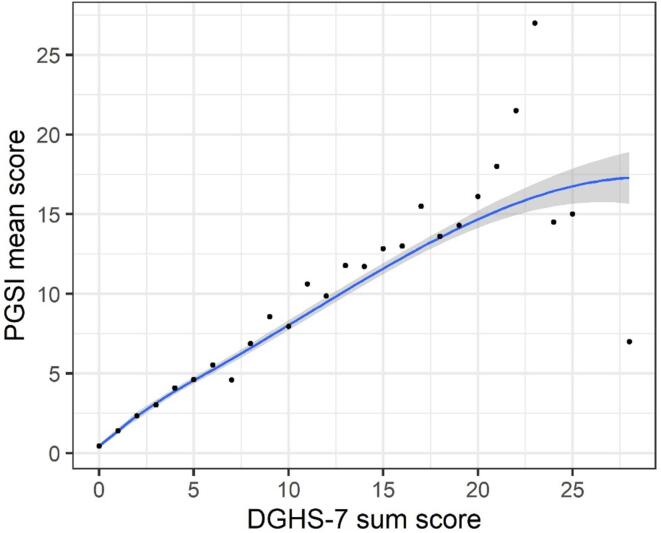
Caption: scatterplot of 7-item domain-general gambling harm scale (dghs-7) sum scores by mean Problem Gambling Severity Index (PGSI) sum scores. Local regression (blue line) is fitted between DGHS-7 sum scores and PGSI sum scores, shaded area depicts 95% confidence interval. (For interpretation of the references to colour in this figure legend, the reader is referred to the web version of this article.)

**Fig. 2:**
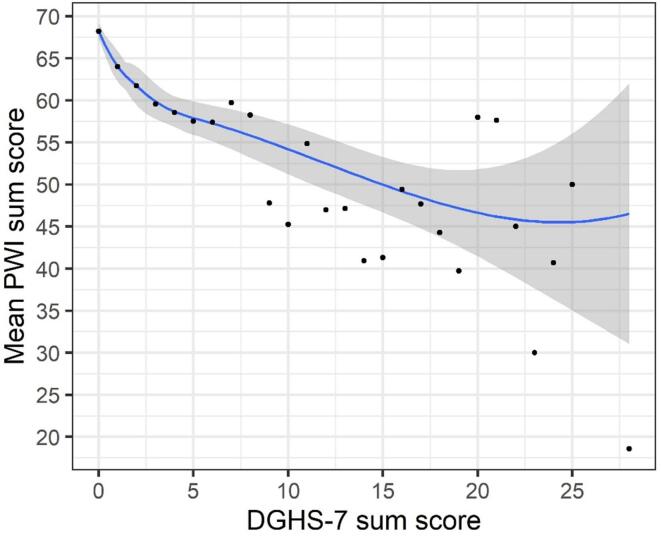
Caption: scatterplot of 7-item domain-general gambling harm scale (dghs-7) sum scores by mean Personal Wellbeing Index (PWI) sum scores. Local regression (blue line) is fitted between DGHS-7 sum scores and PWI sum scores, shaded area depicts 95% confidence interval. (For interpretation of the references to colour in this figure legend, the reader is referred to the web version of this article.)

Table 4 summarizes Spearman’s correlations between composite scores of key study measures. The DGHS-7 correlated strongly with all other gambling measures, including the PGSI (*r_s_* = 0.768), the SGHS (*r_s_* = 0.793), and the full 72-item gambling harms checklist (Harms72; *r_s_* = 0.824). The DGHS-7 also correlated moderately to the PWI (*r_s_* = -0.303). Participants who scored greater than zero on the DGHS-7 (40.4%) had an average PGSI score of 4.3 which was statistically significantly higher than participants not scoring positively on the DGHS-7 (*M* = 0.32; *t*(1264) = 31.167, *p* <.001, Cohen’s *d* = 1.39). Participants who scored positively on the DGHS-7 scored lower on personal wellbeing (*M* = 58.8) compared to participants scoring zero (*M* = 66.3; *t*(2527) = -10.827, *p* <.001, Cohen’s *d* = -0.41). The relationship between DGHS-7 scores and PGSI and PWI scores, respectively, are presented in Fig. 1, Fig. 2. The results indicate that even small increases in DGHS-7 sum scores are associated with higher mean PGSI scores and lower mean PWI scores.

**Table 4:** Spearman correlation matrix.

	DGHS-7	PGSI	SGHS	72-Item checklist
PGSI	0.768^**^	–		
SGHS	0.793^**^	0.755^**^	–	
72-Item checklist	0.824^**^	0.775^**^	0.960^**^	–
PWI	−0.303^**^	−0.265^**^	−0.298^**^	−0.307^**^

Note. Spearman’s correlations. * = Correlation is significant at the 0.05 level (2-tailed). ** = Correlation is significant at the 0.01 level (2-tailed). DGHS-7 = Domain-general gambling harms scale (test-scale). PGSI = Problem gambling severity index. SGHS = Short gambling harm scale. 72-item checklist = Full 72-item gambling harms checklist. PWI = Personal wellbeing index.

Two-tailed independent sample *t*-tests revealed no statistically significant differences concerning the presentation order of the DGHS-7 and the full 72-item gambling harm checklist (DGHS-7 first vs. 72-item checklist first) on the composite score (*p* =.383) and for each individual item except for financial harm which yielded a small but significant difference of which the 72-item checklist first-group reported a higher negative impact (*M* = 0.556, *SD* = 0.844, *n* = 1301) than the DGHS-7 first (*M* = 0.476, *SD* = 0.817, *n* = 1257; *t*(2556) = -2.484, p =.013, Cohen’s d = -0.098). A two-way ANOVA did not reveal a statistically significant interaction effect of presentation order when comparing PGSI-categories on the DGHS-7, *F*(7, 2550) = 2.506, *p* =.057.

## 4 Discussion

The current study investigated the psychometric properties of a 7-item domain-general measure for gambling harm which was derived from a modified version of the 72-item checklist for gambling harm (Li et al., 2017). The DGHS-7 was developed to provide researchers with a brief gambling harm measure that captures the degree of felt impact across each domain of gambling harm.

The results suggest that the DGHS-7 has good fit in terms of a one-factor solution of gambling harms and shows convergent validity with the comprehensive 72-item checklist, the 10-item SGHS, and the PGSI. The results support discriminative validity as the DGHS-7 shows moderate inverse correlation with well-being (PWI). Reliability of the DGHS-7 was also good, as assessed by conventional Cronbach’s alpha, ordinal alpha, and McDonald’s omega (hierarchical). Measurement invariance was supported for gender and income brackets, but it was not possible to compare the originally intended age groups due to low variance in the “law-abidingness” item. This item refers to a type of gambling harm that was originally covered within the “other” domain, typically reflecting gambling harms that are far less frequent in the population and more severe, which could explain why nobody in the 18–29 age group reported “major impact” in relation to this harm (Li et al., 2017). However, we did find support for measurement invariance when comparing those aged 38 and above against those aged below 38 (median split). The aim of DGHS-7 development was construct coverage (i.e., covering all gambling harm domains) which leads to a trade-off in which sensitivity is not maximized for each item. The results also indicated that endorsement of “major impact” of gambling harm was relatively rare across domains, which is in line with previous studies indicating that the most severe harms are experienced by only a minority of individuals (Browne and Rockloff, 2018, Li et al., 2017).

The current study results can be taken together with results from other studies on the 10-item SGHS, the 18-item SGHS, and the 72-item checklist to suggest that gambling harm can be assessed in several ways while still maintaining satisfactory psychometric properties, providing valuable flexibility for studying gambling harm (Browne et al., 2021, Latvala et al., 2021). While prior studies employed concrete present/absent symptomatology at the expense of multi-domain coverage, the present study employed more global Likert measures for each domain of harm. Given these differences, it is notable that we found that the 72-item checklist, the 10-item SGHS, and the DGHS-7 showed similar strength of association with the PGSI and PWI, suggesting that all measures do capture actual harm. This is important because measures of gambling harm must avoid conflating negative consequences from gambling and rational costs of prioritizing gambling over something else—what is termed opportunity cost (Delfabbro et al., 2020). Since economic decisions that act to decrease health or wellbeing are almost certainly not rational, external benchmarks such as the PWI are crucial to establishing that genuine harm is being measured.

As mentioned, the DGHS-7 has a key difference from the 72-item checklist and the SGHS in that it does not provide the respondent with descriptions of specific harms. Rather, the DGHS-7 invites the participant to reflect on an overall category of harm which might be challenging in absence of specific harms prompting recall (although examples were given for law abidingness and cultural harms in the DGHS-7, as these were considered less intuitive categories of harm). This difference motivated the inclusion of a survey experiment investigating possible order effects in answering the 72-item checklist harms before or after answering the domain-general questions. The results largely failed to indicate such order effects, except for a very small increase (small effect size) in reported harms for those answering items about specific financial harms before the general item reflecting financial domain harms, compared to the reverse. The results overall thus support the feasibility of asking respondents to reflect on harms in general. The DGHS-7 also differs from the 72-item checklist and the SGHS by offering a rating scale compared to a binary checklist. In principle, the 5-point rating scale has the potential to be more sensitive to differing degrees of impact, and we feel is certainly appropriate when using a single item to capture an overall degree of impact within a domain. However, given the similar correlations of all measures with the PGSI and the PWI, it would appear that similar degrees of sensitivity can be obtained via either response format (McLauchlan et al., 2020).

There were some limitations to the current study that should be noted. We used Prolific.co to recruit participants. Use of online crowd-working platforms has grown rapidly over recent years which has led to discussion about potential issues with using these services, such as lack of representativeness among participants, quality of participant reports, and demand effects (Bohannon, 2016, Palan and Schitter, 2018). However, Prolific appears to offer diverse samples, subjects that are relatively naïve to research materials (i.e., less chance for demand effects), and attentive during the study (Palan and Schitter, 2018, Peer et al., 2017). In the current survey, we also included an attention check and measured key demographic factors. The median yearly disposable income was £20,000 to £29,000 in the current sample, which was somewhat lower than the median yearly disposable income of £31,400 reported in the 2021 Census in the UK and thus suggesting lack of representativeness in income (Office for National Statistics, 2022). Low representation of high-income brackets also led to the measurement invariance tests for income being limited to a simpler binary grouping. The study also contains several strengths. Notably, the study had a large sample size, and the DGHS-7 was validated against several other scales relevant for convergent (72-item harms checklist, SGHS, PGSI) and discriminant (PWI) validity. The high correlation between the DGHS-7 and the PGSI suggests good convergent validity but can also raise a discussion on the interchangeability of these constructs. Both measures cover problems relating to gambling and the PGSI also measures five gambling harms. However, the PGSI lacks coverage on all seven gambling harm domains which was the main rationale for developing the DGHS-7. Further, there is theoretical and psychometric support for separating the measurement of problem gambling and gambling harm (Browne & Rockloff, 2020). The DGHS-7 was found to have measurement invariance for gender, and age and income brackets when using a median split approach, based on delta CFI value ≤ 0.01 threshold for model comparison (Cheung & Rensvold, 2002). It should be noted that the metric invariance test for gender, residual invariance test for age, and scalar and residual invariance tests for income revealed statistically significant chi-square difference tests, although these tests are susceptible to larger samples making alternative fit indices (such as CFI) more favorable (Putnick & Bornstein, 2016). Finally, certain biases could possibly influence the response styles of participants reporting gambling harms. For instance, individuals with problem gambling can underestimate the costs of gambling due to bounded rationality, which in the current context could lead to reporting less gambling harm than experienced (Fiedler, 2021).

Future studies should expand on the study of the DGHS-7 in several ways. Notably, using a longitudinal design would enable examination of test–retest reliability of the DGHS-7 as well as its predictive validity (for example, whether changes in gambling harms over time are associated with similar changes in wellbeing within subjects). Concerned significant others should also report on the gambling harms they experience through a version of the DGHS-7 adapted for them, as this group has been reported to experience significant distress (Hing et al., 2022, Salonen et al., 2016).

In conclusion, the current study shows support for measuring a wide range of gambling harms with a brief 7-item scale. The DGHS-7 can be useful in instances where researchers experience challenges with survey length, including population-wide studies on gambling and broader studies that assess gambling alongside various other topics. We find similar associations with external benchmarks as reported in prior research on gambling harm using different measurement methods, thus supporting the construct validity of gambling harm, and suggesting that it is not vulnerable to a significant degree of measurement variance.

## Author Note

The present study was funded by the Norwegian Competence Center for Gambling and Gaming Research but received no specific grant.

## Ethics

The current study was exempted from ethical approval in accordance with the Norwegian Centre for Research Data's guidelines for anonymous surveys. Participants were regarded as having provided informed consent by completing the survey.

## CRediT authorship contribution statement

**André Syvertsen:** Conceptualization, Methodology, Data curation, Formal analysis, Writing – original draft, Visualization. **Joakim H. Kristensen:** Conceptualization, Methodology, Data curation, Formal analysis, Writing – review & editing. **Matthew Browne:** Conceptualization, Methodology, Writing – review & editing. **En Li:** Conceptualization, Methodology, Writing – review & editing. **Ståle Pallesen:** Conceptualization, Writing – review & editing, Supervision, Project administration, Funding acquisition.

## Declaration of Competing Interest

The authors declare that they have no known competing financial interests or personal relationships that could have appeared to influence the work reported in this paper.

## Data Availability

Data will be made available on request.
