# Supplementary material for: Validation of the 7-Item Domain-General Gambling Harm Scale (DGHS-7)
Source: Addict Behav Rep. 2023 Jun 2;17:100499. doi: 10.1016/j.abrep.2023.100499 (PMC10279776; doi:10.1016/j.abrep.2023.100499)
Supplement: Supplementary data 1 [file mmc1.docx]

# **‘Validation of the 7-item Domain-General Gambling Harm Scale (DGHS-7)’ Appendix**

**The 7-item Domain-General Gambling Harm Scale (DGHS-7)**

Instruction text: *Please consider your gambling during the last 12 months when answering the following questions.*

Q1: What level of negative impact did your gambling have upon your financial security during this time?

Q2: What level of negative impact did your gambling have upon your personal relationships (family, friends, spouse, partner, etc.) during this time?

Q3: What level of negative impact did your gambling have upon your emotional or psychological wellbeing during this time?

Q4: What level of negative impact did your gambling have upon your physical or mental health during this time?

Q5: What level of negative impact did your gambling have upon your work or study performance during this time?

Q6: What level of negative impact did your gambling have upon your cultural or religious community during this time? (For example, feeling less connected or contributing less to cultural/religious community.)

Q7: What level of negative impact did your gambling have upon your law-abidingness during this time? (For example, taking money or items from friends or family without asking first.)

Response options: 0: No impact; 1: minor impact; 2: some impact 3: moderate impact; 4: major impact
